# The E3 Ubiquitin Ligase SCF Cyclin F Promotes Sequestosome-1/p62 Insolubility and Foci Formation and is Dysregulated in ALS and FTD Pathogenesis

**DOI:** 10.1007/s12035-023-03355-2

**Published:** 2023-05-27

**Authors:** Jennilee M. Davidson, Sharlynn S. L. Wu, Stephanie L. Rayner, Flora Cheng, Kimberley Duncan, Carlo Russo, Michelle Newbery, Kunjie Ding, Natalie M. Scherer, Rachelle Balez, Alberto García-Redondo, Alberto Rábano, Livia Rosa-Fernandes, Lezanne Ooi, Kelly L. Williams, Marco Morsch, Ian P. Blair, Antonio Di Ieva, Shu Yang, Roger S. Chung, Albert Lee

**Affiliations:** 1 Centre for Motor Neuron Disease Research, Macquarie Medical School, Faculty of Medicine, Health and Human Sciences, Macquarie University, Level 1, 75 Talavera Road, Sydney, NSW 2109 Australia; 2 Computational NeuroSurgery (CNS) Lab, Macquarie Medical School, Faculty of Medicine, Health and Human Sciences, Macquarie University, Level 1, 75 Talavera Road, Sydney, NSW 2109 Australia; 3 Illawarra Health and Medical Research Institute, Northfields Avenue, Wollongong, NSW 2522 Australia; 4 School of Chemistry and Molecular Bioscience and Molecular Horizons, University of Wollongong, Northfields Avenue, Wollongong, NSW 2522 Australia; 5 Centro de Investigación Biomédica en Red de Enfermedades Raras (CIBERER U-723), Unidad de ELA, Instituto de Investigación Hospital 12 de Octubre de Madrid, SERMAS, Madrid, Spain; 6 Neuropathology Department and CIEN Tissue Bank, Alzheimer’s Centre Reina Sofia-CIEN Foundation, 28031 Madrid, Spain

**Keywords:** Cyclin F, Sequestosome-1/p62, Ubiquitylation, Aggregation, Amyotrophic lateral sclerosis, Frontotemporal dementia

## Abstract

Amyotrophic lateral sclerosis (ALS)- and frontotemporal dementia (FTD)-linked mutations in *CCNF* have been shown to cause dysregulation to protein homeostasis. *CCNF* encodes for cyclin F, which is part of the cyclin F-E3 ligase complex SCF^cyclinF^ known to ubiquitylate substrates for proteasomal degradation. In this study, we identified a function of cyclin F to regulate substrate solubility and show how cyclin F mechanistically underlies ALS and FTD disease pathogenesis. We demonstrated that ALS and FTD-associated protein sequestosome-1/p62 (p62) was a canonical substrate of cyclin F which was ubiquitylated by the SCF^cyclinF^ complex. We found that SCF^cyclin F^ ubiquitylated p62 at lysine(K)281, and that K281 regulated the propensity of p62 to aggregate. Further, cyclin F expression promoted the aggregation of p62 into the insoluble fraction, which corresponded to an increased number of p62 foci. Notably, ALS and FTD-linked mutant cyclin F p.S621G aberrantly ubiquitylated p62, dysregulated p62 solubility in neuronal-like cells, patient-derived fibroblasts and induced pluripotent stem cells and dysregulated p62 foci formation. Consistently, motor neurons from patient spinal cord tissue exhibited increased p62 ubiquitylation. We suggest that the p.S621G mutation impairs the functions of cyclin F to promote p62 foci formation and shift p62 into the insoluble fraction, which may be associated to aberrant mutant cyclin F-mediated ubiquitylation of p62. Given that p62 dysregulation is common across the ALS and FTD spectrum, our study provides insights into p62 regulation and demonstrates that ALS and FTD-linked cyclin F mutant p.S621G can drive p62 pathogenesis associated with ALS and FTD.

**Supplementary Information:**

The online version contains supplementary material available at 10.1007/s12035-023-03355-2.

## Introduction

Amyotrophic lateral sclerosis (ALS) and frontotemporal dementia (FTD) are fatal, progressive neurodegenerative diseases. ALS and FTD display both clinical and genetic overlap and are increasingly recognized to exist on a disease spectrum. Patients can present with either inherited (familial) or sporadic ALS and/or FTD. In ALS and FTD, motor neurons undergo progressive cell death, which is characterized by the hallmark accumulation of protein aggregates in the brain or spinal cord [1].

Several missense mutations have been identified in the *CCNF* gene in patients with ALS and/or FTD [2, 3]. Cyclin F (encoded by the *CCNF* gene) [NM_001761.3, NP_001752.2] is a substrate adaptor of the RING finger E3 ubiquitin ligase Skp1-Cul1-F-box complex (SCF complex) [4], which ubiquitylates substrates typically for degradation by the ubiquitin proteasome system (UPS) [5]. Cyclin F is a 786 amino acid protein that has an N-terminal catalytic F-box domain, a substrate recruitment Cyclin Box domain, a C-terminal PEST domain, and two nuclear localisation signals [5]. Within the Cyclin Box domain lies the hydrophobic ^309^MRYIL^313^ sequence (amino acids Met-Arg-Tyr-Ile-Leu) that is important for substrate binding. This MRYIL sequence is conserved across various mammalian cyclins [6] and interacts with substrates containing cyclin-binding (CY) motifs (amino acids Arg-X-Leu/Ile or RxL/I) (where X is any amino acid) [4, 7]. Since cyclin F is integral to the UPS, most studies have focused on the mechanistic role of cyclin F mediating the proteasomal degradation of its substrates.

Cyclin F is tightly regulated throughout the cell cycle and as such cyclin F substrates are predominantly reported to be involved in cell cycle progression and maintaining genomic stability. These substrates include nucleolar and spindle-associated protein 1 (NuSAP1) [8], cell division cycle 6 (CDC6) [9], stem-loop binding protein (SLBP) [10], ribonucleotide reductase M2 (RRM2) [11], centriolar coiled coil protein 110 (CP110) [7, 8], transcription factors (E2Fs) [12–14], exonuclease 1 (EXO1) [15], cadherin-1 (Chd1/fzr1) [16], recombinant signal binding protein for immunoglobulin kappa J region (RBPJ) [17], and NAD-dependent protein deacylase sirtuin-5 (SIRT5) [18]. Given most neurons reside in the post-mitotic, or G1, phase the functional role of cyclin F in neurons and ALS and FTD pathogenesis remains unclear.

Serine to glycine mutation (p.S621G) is the most studied *CCNF* mutation to date since this mutation segregates well with disease across generations [2]. Our team previously identified that the cyclin F p.S621G mutation causes an increase in its E3 ubiquitin ligase activity for Lysine(K)48-linked polyubiquitylation [19–21] which leads to the accumulation of ubiquitylated ALS-associated protein TDP-43 and SCF^cyclin F^ target protein RRM2 in neuronal cells [2]. Notably, we recently reported that mutant p.S621G aberrantly K48-ubiquitylates TDP-43 causing its accumulation in neurons; an aberrant mechanism that likely contributes to the skein-like cytoplasmic TDP-43 aggregates in cyclin F p.S195R patient tissue [22]. Ultimately, expression of mutant cyclin F p.S621G leads to altered proteostasis of its interaction partners [22, 23], defects in protein degradation systems [20], and the activation of cell death pathways [21, 24]. Cyclin F also interacts with ALS-associated proteins VCP (valosin-containing protein) [25] and sequestosome-1/p62 (p62) [20, 25]. These data strengthen the pathogenetic link between cyclin F mutations, notably p.S621G, to ALS and FTD pathogenesis [26]. Whether p62 is a substrate of the E3 ubiquitin ligase SCF ^cyclin F^ complex remains unclear. Additionally, how the mutant cyclin F p.S621G-p62 interaction contributes to ALS and FTD pathogenesis is also unclear but may relate to defective autophagy and protein clearance [2, 20].

Dysregulated p62 levels and aggregates of p62 have been documented in sporadic and familial ALS and/or FTD cases, including cellular inclusions in the brain and the spinal cord [27–30]. Genetically linked variants of *SQSTM1*/p62 directly implicates p62 in the disease pathogenesis and offers insight into how altered p62 proteostasis may increase the susceptibility to neurodegeneration [29, 31–33]. p62 is a multifunctional protein involved in several signalling pathways including selective autophagy, inclusion body formation, protein quality control and various cellular signalling like antioxidant and anti-inflammatory pathways, which have all been implicated in ALS and FTD pathogenesis [34, 35]. The varied functions of p62 are regulated through post-translational modifications [36–38]. Phosphorylation of p62 regulates p62 recognition and capture of ubiquitylated substrates for autophagic clearance [39–42]. Although ubiquitylation is typically associated to degradation pathways, ubiquitylation of p62 produces diversified effects on its functions. Ubiquitin ligases can inhibit or activate p62 function depending on the site of ubiquitylation [43–46]. It has been found that the ubiquitylation of p62 mediates p62 inclusion body formation for autophagy under basal conditions [46], which are different from p62 positive pathological aggregates at the end stage of neurodegenerative diseases. However, how the aberrant ubiquitylation activity of mutant cyclin F regulates p62 remains unknown.

In this study we investigated the possible role of cyclin F-mediated regulation of p62 in ALS and FTD pathogenesis. We found that cyclin F, as part of the SCF ubiquitin ligase complex, interacts with p62 through the MRYIL sequence in the Cyclin Box domain, and that p62 was recognized in a CY motif-dependent manner. We report that p62 is a substrate of cyclin F for SCF^cyclin F^-mediated ubiquitylation at K281, which regulated p62 solubility. Cyclin F expression also promoted p62 to form an increased number of p62 foci which corresponded to the aggregation of insoluble p62. Notably, the ALS and FTD-linked mutant cyclin F p.S621G abnormally ubiquitylated p62, reduced the aggregation of insoluble p62 and reduced p62 foci formation. Our studies demonstrate that the E3 ubiquitin ligase SCF^cyclin F^ complex functions in p62 proteostasis and that ALS and FTD-linked cyclin F mutant p.S621G can drive p62 pathogenesis related to neurodegeneration.

## Results

### Cyclin F Interacts with Sequestosome-1/p62

**Fig. 1:**
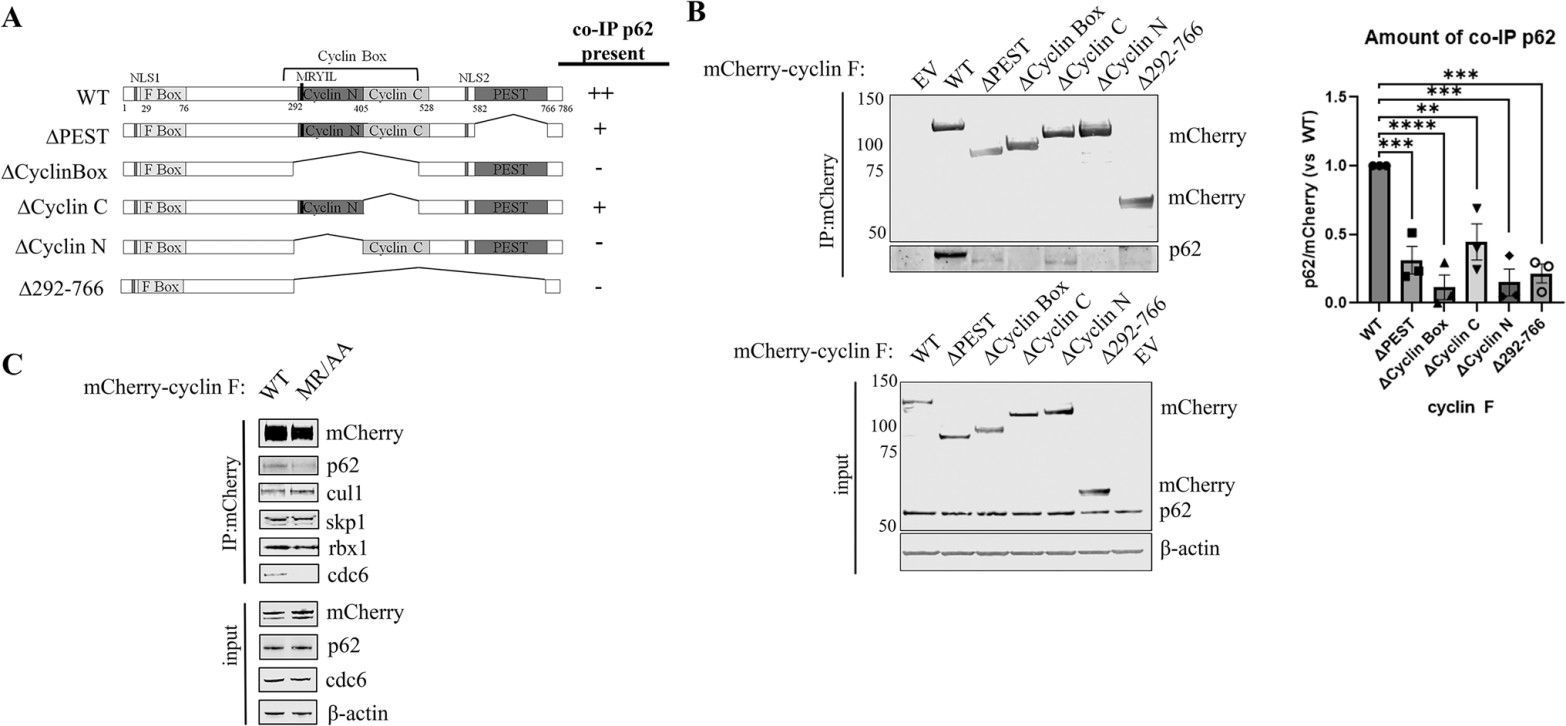
Cyclin F binds p62 via MRYIL sequence. **A**. Schematic representation of mCherry-cyclin F WT and deletion mutants examined for their interaction with endogenous p62. Presence of endogenous co-immunoprecipitated p62 on immunoblot is indicated with ‘ + ’. **B**. Lysates prepared from HEK293 cells transiently expressing mCherry-cyclin F WT or deletion mutants (input) were subjected to immunoprecipitation using RFP Trap magnetic beads recognizing the mCherry tag on cyclin F (*n* = 3) to identify the region required for interacting with p62. Immunoblot analysis was performed using the indicated antibodies. The amount of co-immunoprecipitated p62 was normalized to the amount of immunoprecipitated mCherry-cyclin F prior to comparing to WT **C.** Lysates prepared from HEK293 cells transiently expressing mCherry-cyclin F WT or MRYIL mutant (MR/AA) were subjected to immunoprecipitation using RFP Trap magnetic beads recognizing the mCherry tag on cyclin F followed by immunoblot analysis using the indicated antibodies. Endogenous p62 co-immunoprecipitated with cyclin F WT but was diminished in Cyclin F MR/AA mutant eluate (*n* = 3). IP, immunoprecipitation. NLS, nuclear localization signal

Cyclin F and p62 co-immunoprecipitate in Neuro2A and HEK293T cells [20, 25]. To delineate the region of p62 that mediates interaction with cyclin F, we expressed a series of cyclin F deletion mutants in HEK293 cells and immunoprecipitated the different cyclin F mutants to assess their ability to co-immunoprecipitate with endogenous p62 (Fig. 1A and B). We found that deletion of the N-terminal region of the Cyclin Box domain (∆CyclinN) and deletion of the Cyclin Box (∆CyclinBox) disrupted its binding to p62 (Fig. 1B), indicating that this region is responsible for the association with p62. The binding of cyclin F to p62 was also attenuated by the deletion of the 292-766 region (∆292-766), and this attenuation was partially reversed by addition of the Cyclin N domain (∆PEST and ∆Cyclin C). The ∆292–766 construct had negligible binding, which may be ascribed to the generation of the cyclin F deletion construct that connected similar amino acids from the C-terminal region (amino acids 766–786) to the deleted amino acids of the cyclin box without a linker region. These results suggested that the N-terminal region of the Cyclin Box domain facilitates interaction with p62.

The Cyclin Box domain within cyclin F contains a hydrophobic patch motif [4, 7]. This hydrophobic patch amino acid sequence MRYIL associates with the cyclin-binding motif in its substrates [7, 11]. We next investigated whether p62 is recognized via the MRYIL sequence of cyclin F given its location within the N-terminal region of the Cyclin Box (CyclinN region). Mutations in the MRYIL sequence have previously been used to identify protein interactors such as CDC6 [9], B-Myb [47], RRM2 [11], E2F1 [13], CP110 [7]. We generated a mCherry-cyclin F (p.M309A/R310A referred to as MR/AA) mutation to disrupt the MRYIL binding sequence (^309^MRYIL/AAYIL^313^) and expressed the cyclin F (MR/AA) mutant in HEK293 cells for 24 h followed by co-immunoprecipitations (Fig. 1C). We observed the cyclin F MR/AA mutant had retained binding to the SCF complex components, Skp1, Cul1, Rbx1, confirming that the cyclin F MR/AA mutant does not disrupt the SCF complex formation, while losing most of its binding to endogenous p62 and known protein interactor CDC6 [9] (Fig. 1C). This confirmed that cyclin F recognizes p62 through its MRYIL sequence.

**Fig. 2:**
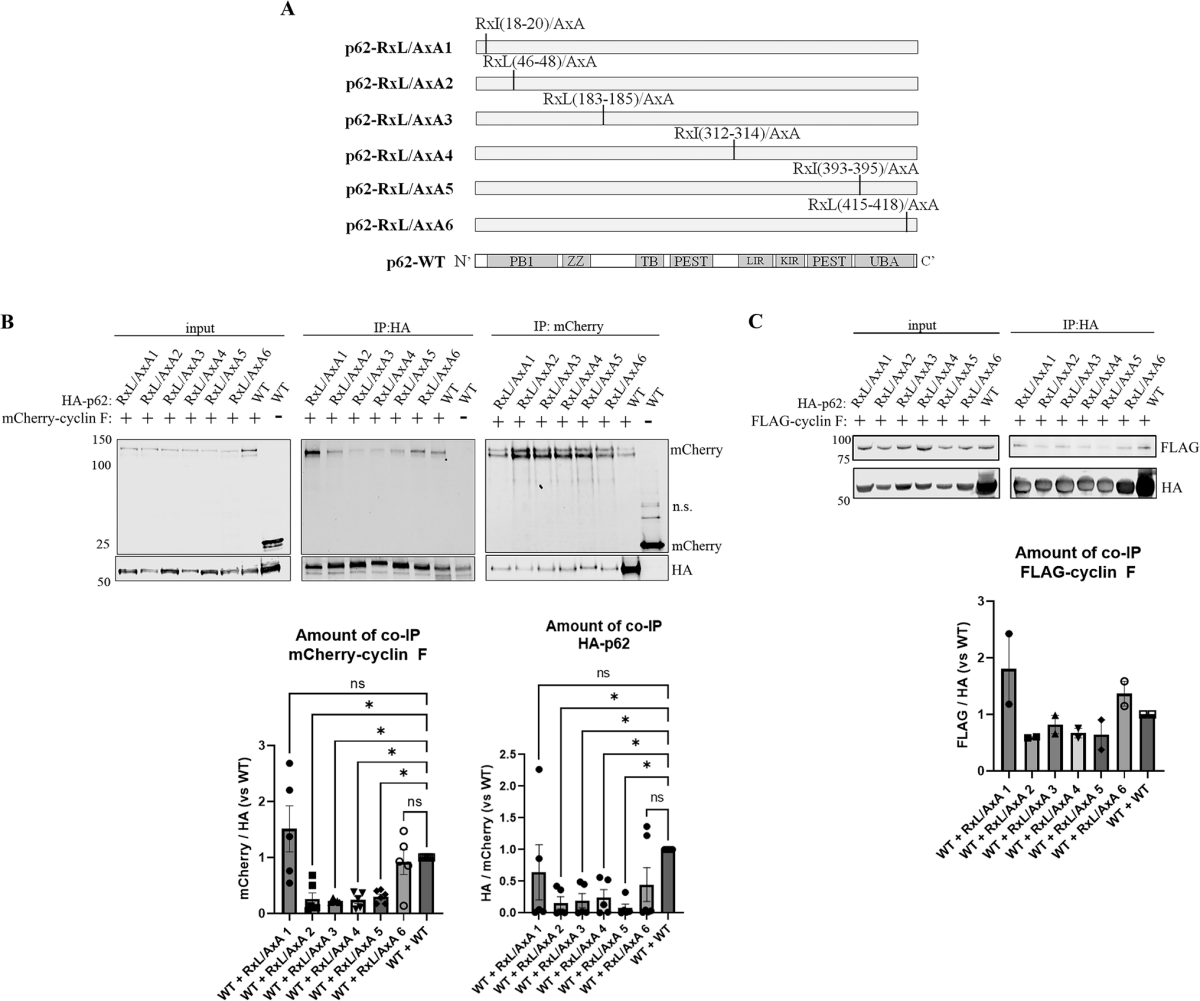
p62 interacts with cyclin F in a CY-motif dependent manner. **A**. Schematic representation of individual HA-tagged p62 CY mutants and WT that were co-expressed with cyclin F in HEK293 cells. p62 was immunoprecipitated (IP) with magnetically coupled HA-antibody, lysates (input) and IP were analyzed by immunoblot. **B** Immunoblot of co-immunoprecipitated mCherry-cyclin F with p62 CY mutants (*n* = 5) showing significantly disrupted co-interaction of cyclin F with p62 when four CY motifs were mutated individually (RxL/AxA 2–5). The amount of co-IP mCherry-cyclin F normalized to the amount of IP HA-p62 was quantified to confirm significantly loss of co-interaction due to CY motifs 2–5 (below IP:HA immunoblot). Reverse IP confirmed disrupted co-interaction of p62 CY (RxL) mutants 2–5 (*n *= 5). The amount of co-IP HA-p62 normalized to the amount of IP mCherry-cyclin F was quantified to confirm significantly loss of co-interaction due to CY motifs 2–5 (below IP:mCherry immunoblot). **C.** Immunoblot confirming disrupted co-IP FLAG-cyclin F with p62 CY mutants (RxL/AxA 2–5) (*n *= 2). Quantification of co-immunoprecipitated FLAG-cyclin F normalized to immunoprecipitated HA is shown below immunoblot. n.s., not specific

Cyclin F associates with its substrates via recognition of the CY motif sequences (amino acids R-X-L/I) within these substrates. Notably p62 contains six putative CY motifs. Given the MRYIL sequence of cyclin F influenced its interaction with p62, we hypothesized that p62 was a canonical substrate that was recognized by its CY motif. To test this, we constructed several mutants of p62, each deficient of one of its CY motifs by mutating the RxL or RxI sites to AxA: RxI(18–20)/AxA, RxL(46–48)/AxA, RxL(183–185)/AxA, RxI(312–314)/AxA, RxI(393–395)/AxA, RxL(415–418)/AxA, now termed p62-RxL/AxA 1–6, respectively (Fig. 2A). These constructs were co-transfected with mCherry-tagged cyclin F or mCherry-tag only in HEK293 cells. After 24 h, the cells were lysed and HA-tagged p62 was immunoprecipitated from cell lysates and analyzed using immunoblot analysis. We found that mutation of four of the individual CY motifs (RxL/AxA 2–5) disrupted binding to cyclin F, indicating that one or more RxL sites are required for the association of p62 with cyclin F (Fig. 2B). To confirm this CY motif-dependent interaction, we used the same lysate and conducted a reverse IP using RFP Trap to enrich mCherry-tagged cyclin F or mCherry-tag only and immunoblotted for HA-tagged p62. Consistently, we found that mutation of the RxL sites disrupted the binding to cyclin F (Fig. 2B). The difference in binding affinities may be ascribed to the reverse co-immunoprecipitation identifying only a sub quantity of the CY motif mutants. To further confirm these findings, we repeated the experiment under the same conditions instead immunoprecipitating FLAG-tagged cyclin F that was co-expressed with each of the HA-tagged p62 RxL/AxA mutants, confirming that the tag itself does not influence substrate binding. Indeed, we found that cyclin F interaction with p62 was disrupted by the RxL/AxA mutations (Fig. 2C). Together these experiments confirmed that p62 interacts with cyclin F in a CY motif-dependent manner.

**Fig. 3:**
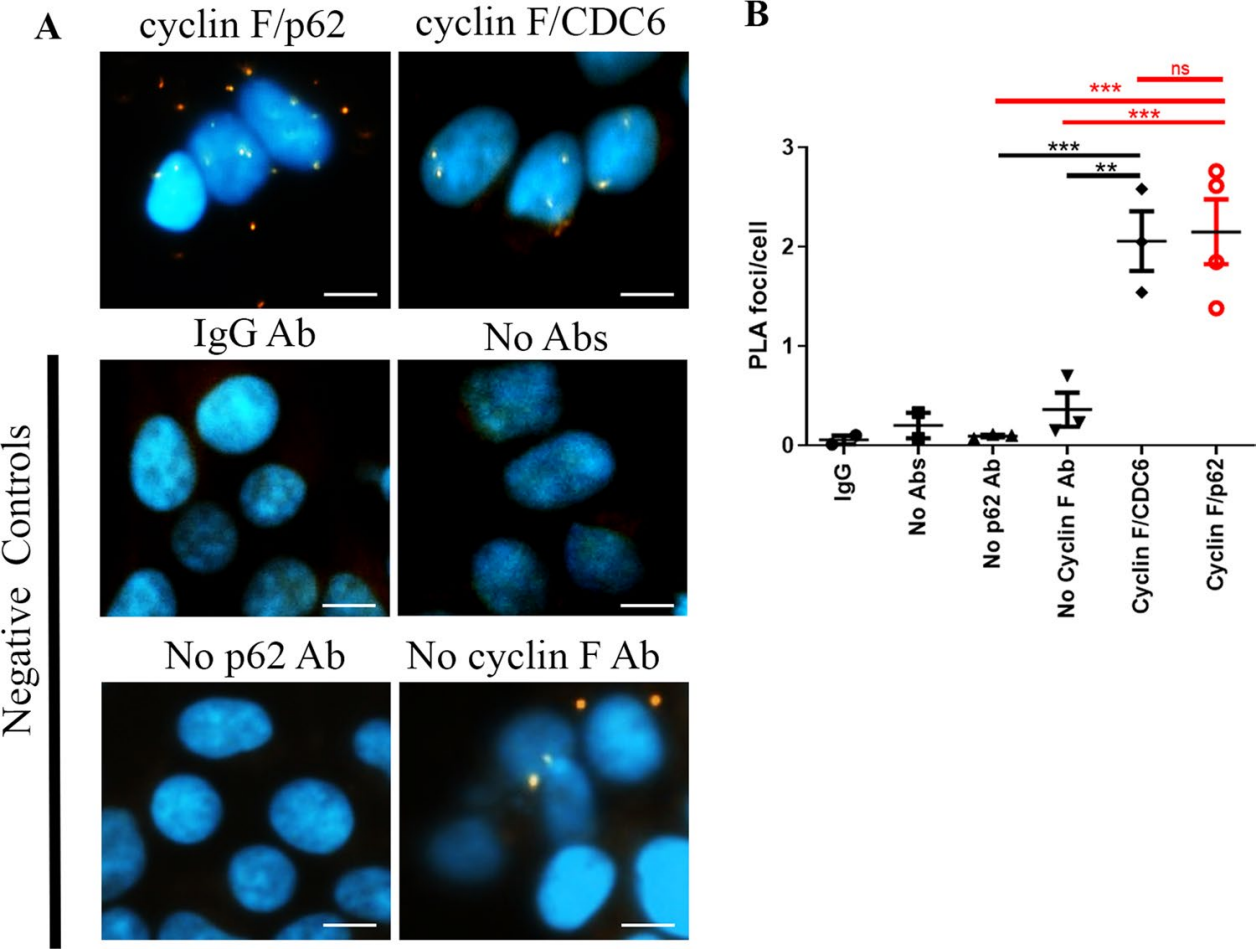
Endogenous cyclin F and p62 co-localize. **A**. PLA analysis of endogenous protein interaction in HEK293 cells using antibodies against cyclin F and p62 together, cyclin F and cdc6 (positive control), and negative controls cyclin F or p62 alone, control IgG or no primary antibodies. Red foci are indicative of sites of protein interaction. DAPI was used for nuclear staining (blue). Scale bar represents 10 µm. **B**. Quantification of mean ± SEM number of foci per cell for each PLA condition. The number of foci in each PLA analysis were compared to cells co-stained with cyclin F and p62 antibodies by one-way ANOVA with Brown-Forsythe correction for unequal variance and Dunnett’s multiple comparison post-hoc test used for statistical analysis. In technical negative controls, which were cells stained with no primary antibodies, we observed a negligible number of foci (*p* < 0.005). Performing PLA with either cyclin F or p62 antibody alone produced a low number of foci (*p* < 0.0005 and *p* < 0.0005). Staining with only mouse IgG antibody, we observed a negligible number of foci (*p* < 0.005). Experimental condition for cyclin F-p62 interaction shown in red. **p < 0.005, ***p < 0.0005, ns not significant

Next, to confirm that endogenous cyclin F and p62 colocalized, we used an *in-situ* proximity ligation assay (PLA), which allows for the detection of low abundance proteins. PLA utilizes rolling circle DNA amplification that can be visualized by fluorescently labelled complementary oligonucleotide probes. The result is fluorescent foci at spots of < 40 nm proximity (the endogenous protein interaction) that can be visualized by fluorescence microscopy [48]. For PLA analysis, the number of fluorescent foci were calculated using a python script (https://github.com/doc78/PLA-Analysis). PLA analysis revealed an unambiguous co-localization of cyclin F and p62 upon fluorescence complementation. The number of fluorescent foci in cells stained for cyclin F and p62 was significantly increased by 20-fold and four-fold compared to cyclin F or p62 antibody alone, respectively (Fig. 3A). Co-staining with cyclin F and CDC6 antibodies served as a positive control since cyclin F and CDC6 are known protein interactors [9]. PLA performed on cells stained with cyclin F and CDC6 antibodies together produced a similar number of foci compared to co-stained cells with cyclin F and p62 antibodies together (Fig. 3B). A significantly greater number of foci was also detected for cyclin F and CDC6 stained cells compared to cyclin F or p62 antibodies alone. Together, these data indicated that endogenous cyclin F and p62 co-localize in close proximity and confirms together with our previous results that cyclin F and p62 are protein interactors.

### Cyclin F Promotes the Ubiquitylation of p62

**Fig. 4:**
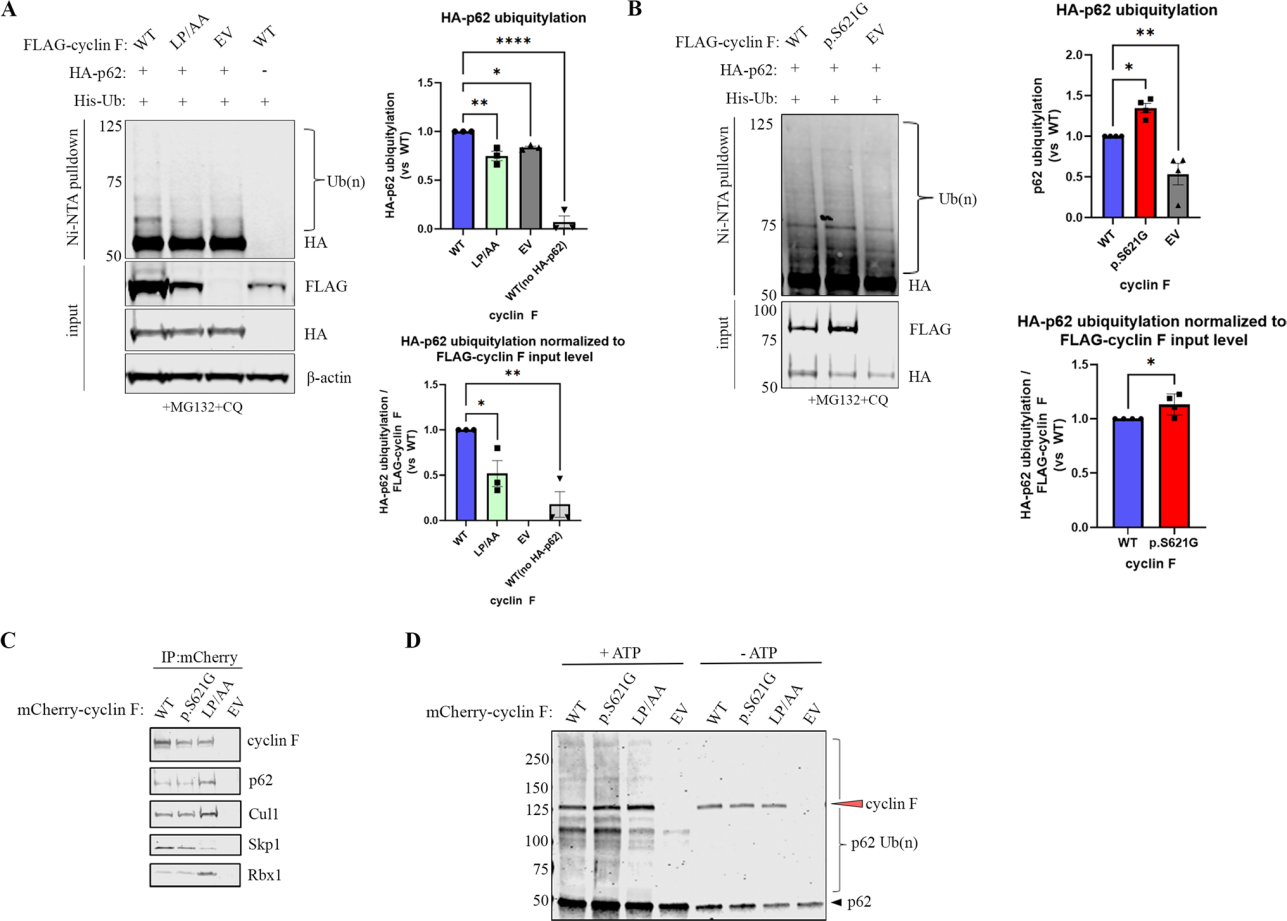
Cyclin F promotes the ubiquitylation of p62. In vivo ubiquitylation assay was carried out by co-expressing His-tagged ubiquitin construct together with FLAG-tagged cyclin F (WT, LP/AA, p.S621G) or FLAG-EV and our potential substrate, HA-tagged p62, in the presence of MG132 and chloroquine (CQ), in HEK293 and Neuro2A cells (input). Immunoprecipitation (IP) was conducted under denaturing conditions. Immunoblot analysis revealed that **A**. the addition of FLAG-cyclin F WT resulted in higher molecular weight HA signal in the Ni–NTA pulldown in HEK293 cells, indicating p62 was ubiquitylated in the presence of cyclin F (*n* = 3). Quantification showing cyclin F WT expression resulted in significantly more p62 ubiquitylation (top panel graph), which was consistent when the level of p62 ubiquitylation was normalized to the level of FLAG-cyclin F expression (bottom panel graph). **B**. p62 was more ubiquitylated in the presence of cyclin F p.S621G in Neuro2A cells (*n *= 3). Quantification showing cyclin F p.S621G expression resulted in significantly more p62 ubiquitylation (top panel graph). The level of p62 ubiquitylation was slightly greater, yet still significant, when ubiquitylation level was normalized to the level of FLAG-cyclin F expression (bottom panel graph). **C.** HEK293 cells transiently expressing mCherry-tagged cyclin F WT, p.S621G, LP/AA or EV were subjected to immunoprecipitation for the in vitro ubiquitylation assays (*n* = 5). **D**. In vitro ubiquitylation assays were conducted with IP cyclin F WT, p.S621G, LP/AA or mCherry-alone, recombinant fragment of p62, E1 and E2 conjugating enzymes, biotinylated-ubiquitin, with ( +) and without (-) ATP (*n* = 4) and then analyzed by immunoblot with the indicated antibodies. Black arrow indicates monomeric (or fragment) p62. Red arrow indicates cyclin F probing after p62 probing to confirm absence or presence of cyclin F in the reaction and confirms consistent amounts of immunoprecipitated cyclin F were compared across experimental conditions. Dark smears and higher molecular weight bands indicate ubiquitylated forms of p62. Ub(n), ubiquitylation

Cyclin F functions as a substrate adaptor in the protein SCF^cyclin F^ ubiquitin ligase complex. Thus, we next questioned whether cyclin F regulates the ubiquitylation of p62. We co-expressed a His-tagged ubiquitin construct together with FLAG-tagged cyclin F variants or FLAG-EV and our potential substrate, HA-tagged p62, in the presence of MG132 and chloroquine (CQ) in Neuro2A and HEK293 cells. Notably, the HA tag does not contain lysine sites for ubiquitylation. To confirm ubiquitin was covalently attached to p62, we performed immunoprecipitation using a nickel charged resin in the presence of 7 M urea. We found that the presence of FLAG-cyclin F WT promoted p62 ubiquitylation in HEK293 cells as indicated by the higher molecular weight species of HA signal (Fig. 4A). Cyclin F LP/AA, which maintains binding to substrates but leads to a reduction in ubiquitylation of substrates [7, 9, 22], as well as EV consistently resulted in a lower amount of ubiquitylated of p62 compared to WT (Fig. 4A). This indicates that cyclin F regulates p62 ubiquitylation.

To evaluate the effect of the ALS and FTD p.S621G mutation on the ubiquitylation of p62, we also conducted ubiquitylation assays with the addition of cyclin F p.S621G in Neuro2A cells. In previous studies, we have shown that the cyclin F p.S621G mutation leads to an increase in the ubiquitylation of substrates [19, 22]. Notably, the presence of cyclin F p.S621G led to greater ubiquitylation of p62 compared to WT (Fig. 4B). There was a slight, yet significant, level of p62 ubiquitylation after normalizing the p62 ubiquitylation to the level of FLAG-cyclin F, which is likely due to the transient co-expression used for this assay. Together, these data suggest that cyclin F p.S621G leads to aberrant ubiquitylation of p62 in cell culture.

Next, we investigated whether p62 was an ubiquitylation substrate of SCF^cyclin F^ by utilizing an in vitro ubiquitylation assay that combined all the components required in vitro. In this experiment we also investigated the effect of the cyclin F p.S621G ALS and FTD variant on the ability of cyclin F to ubiquitylate p62. mCherry-cyclin F variants were immunoprecipitated from cell lysates to ensure the SCF complex was intact, which maintains its enzymatic activity with the mCherry-tag [19]. Purified recombinant p62 was incubated with biotinylated-ubiquitin and immunoprecipitated cyclin F in the presence of E1 (UBA1) and E2 (UBE2D3) conjugating enzymes, with and without ATP, and then analyzed by immunoblot. The recombinant p62 used was a commercially available fragment containing amino acids 85–422 of p62, which contains 15 of 17 the known ubiquitylation sites.

Immunoprecipitation of cyclin F WT, ALS and FTD mutant p.S621G, and LP/AA confirmed the interaction with p62, which was not observed with the EV alone (Fig. 4C). Skp1, Cul1 and Rbx1 all co-immunoprecipitated with both cyclin F WT and p.S621G which indicated that the SCF complex was intact for the in vitro ubiquitylation assay whilst the LP/AA variant had reduced binding to Skp1 (Fig. 4C). Our results suggest that recombinant p62 was ubiquitylated by cyclin F as a part of the SCF^cyclin F^ complex (Fig. 4D). Cyclin F LP/AA consistently resulted in less ubiquitylation of p62 compared to WT, and in the absence of ATP, we did not detect any ubiquitylation activity on p62. Consistently, the ALS and FTD disease causing cyclin F p.S621G variant aberrantly ubiquitylated p62 (Fig. 4D). Together, these results confirm that p62 is a ubiquitylation substrate of SCF^cyclin F^ and that cyclin F p.S621G aberrantly ubiquitylates p62.

### p62 in Cyclin F p.S621G and p.S195R Patients is Hyperubiquitylated

**Fig. 5:**
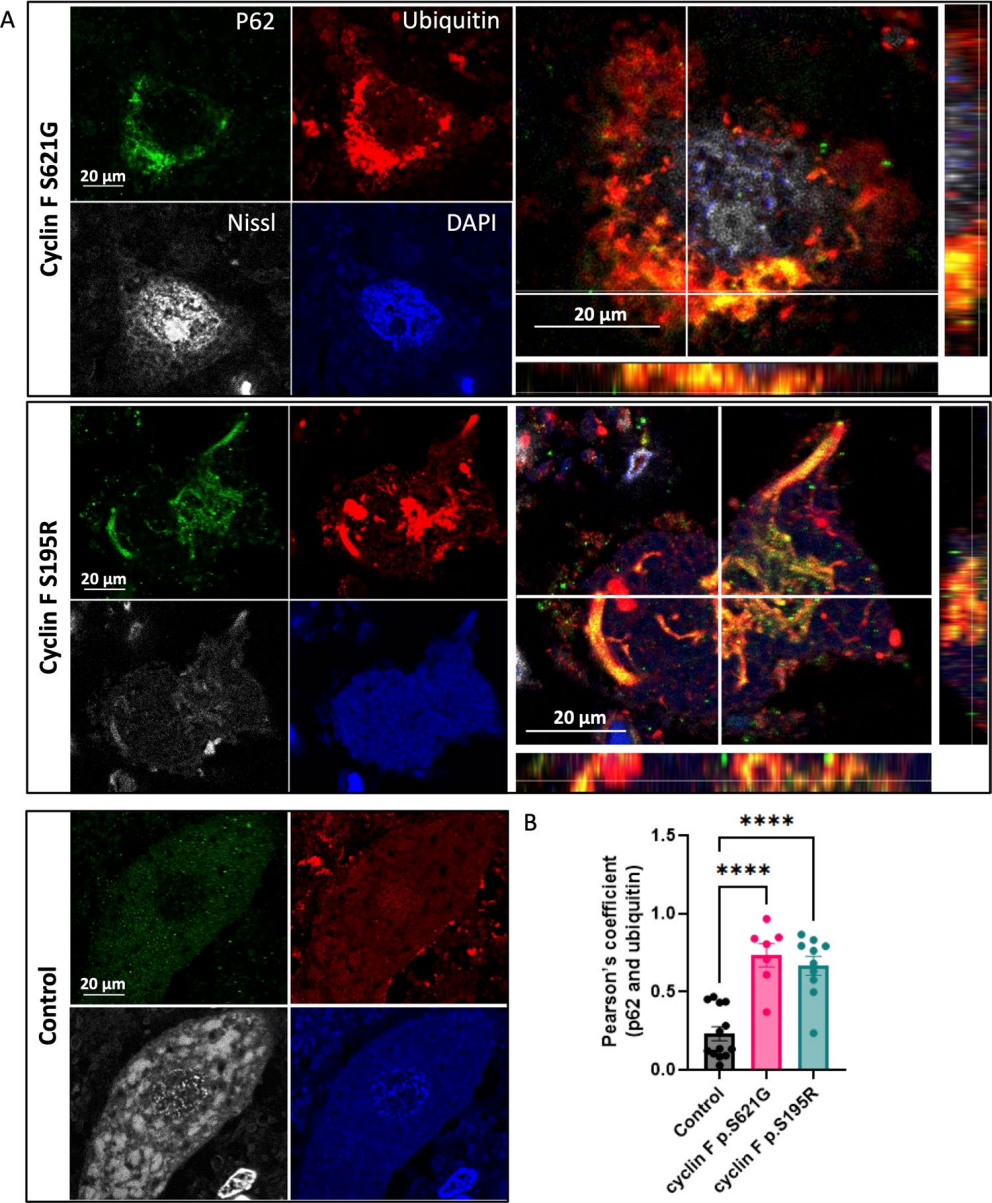
p62 is hyperubiquitylated in cyclin F patient post-mortem spinal cord motor neurons. **A**. Immunofluorescence of p62 (green) and ubiquitin (red) in a healthy motor neuron from control or degenerating motor neurons from cyclin F patient spinal cord tissue sections. Enlarged images showed orthogonal views of p62 and ubiquitin staining. **B**. Areas of p62 and ubiquitin inclusion were obtained from Fiji Image J colocalization analysis plugin BIOP JaCoP. Cyclin F p.S621G and p.S195R patient showed significantly greater co-localized ubiquitin and p62 compared to controls, suggesting hyperubiquitylation of p62. Scale bar in white. ****p < 0.0001

To validate in patient tissue, we performed immunofluorescence analysis on post-mortem spinal cord tissue from patients with cyclin F mutations (p.S621G and p.S195R) and controls. Given that we previously demonstrated that cyclin F p.S195R mutation leads to a significant increase in SCF^cyclin F^ E3 ubiquitylation activity [21], and that its overexpression leads to an accumulation of ubiquitylated substrates [2], we hypothesized that aberrant ubiquitylation of p62 may also occur in patients with the cyclin F p.S195R mutation and performed immunofluorescence analysis on p.S195R post-mortem spinal cord tissue as well. As shown in Fig. 5, we found large neuronal inclusions that are strongly positive for both p62 and ubiquitin in all cyclin F patient tissues but not in control. In cyclin F patients, we observed that p62 inclusions were found to be largely colocalized into ubiquitin but ubiquitin inclusions were not necessarily positive for p62. Accordingly, we found that ubiquitin colocalized significantly more with p62 in patient tissue compared to the controls (ANOVA with Dunnett’s multiple comparisons test of Pearson’s correlation,* p* < 0.0001 for p.S621G and p.S195R tissue) indicating hyperubiquitylation of p62 at end stage of disease in cyclin F-linked patient tissue.

### Cyclin F-Mediated Ubiquitylation Site Lysine 281 on p62 Regulates Solubility

**Fig. 6:**
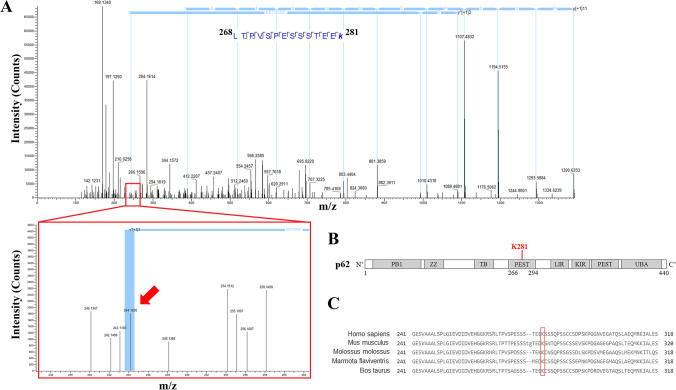
Cyclin F ubiquitylates lysine 281 of p62. **A**. MS/MS spectra of in vitro ubiquitylation of recombinant p62 by immunoprecipitated cyclin F from HEK293 cells identified lysine281 to be ubiquitylated. MS/MS spectra of the corresponding p62 peptide ^268^LTPVSPESSSTEEK^281^ with the C-terminal lysine (K) 281 containing the ubiquitin adduct. **B**. Schematic of cyclin F-mediated K281 ubiquitylation site on p62. **C**. Cyclin F ubiquitylated recombinant p62 in vitro at conserved K281 site

To identify the cyclin F-mediated ubiquitylation site on p62, we analyzed the in vitro ubiquitylation assays by mass spectrometry (MS) [49]. We found that p62 was ubiquitylated by all cyclin F variants at a previously reported ubiquitylation site K281 [50], which was not ubiquitylated by the empty vector control (Fig. 6A and B). Moreover, protein sequence analysis of the p62 region encompassing the ubiquitylation site (amino acids 241–318) suggests that K281 site is conserved among species (Fig. 6C).

**Fig. 7:**
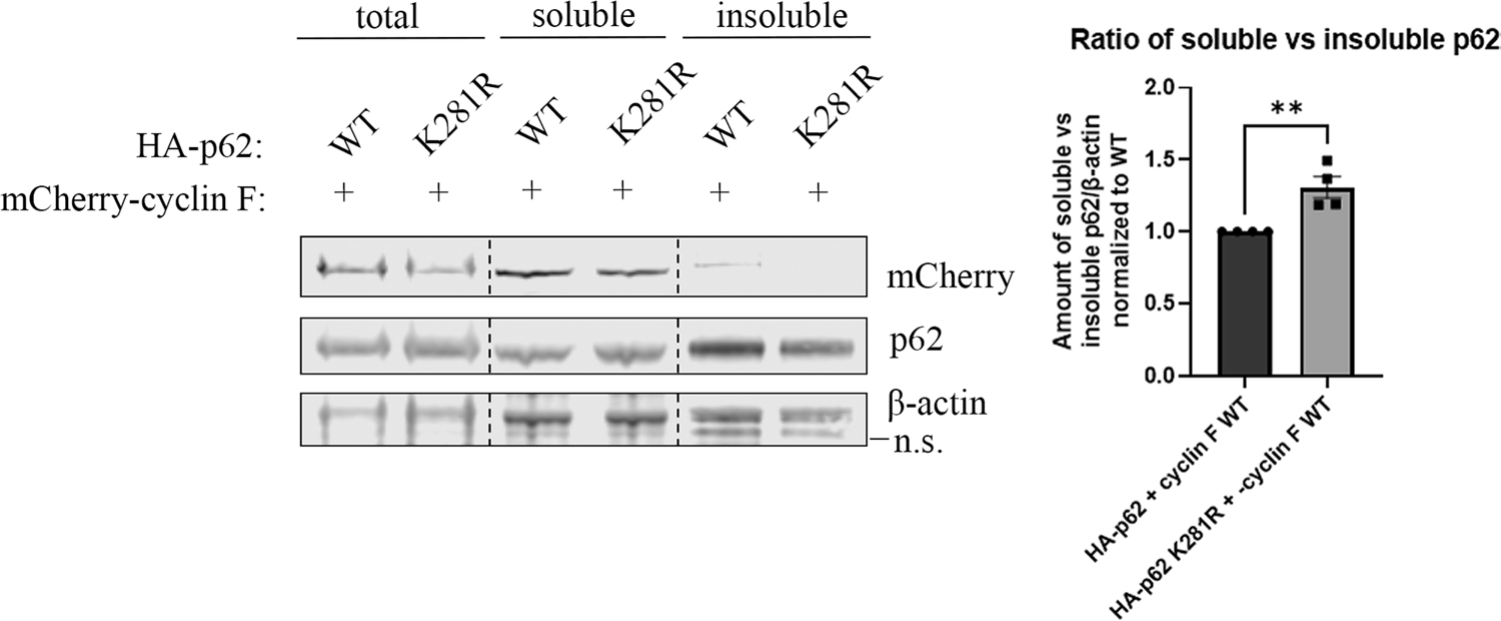
Cyclin F-mediated ubiquitylation site K281 regulates p62 solubility. **A**. NSC-34 cells transiently co-expressing combinations of HA-tagged p62 WT or K281R mutant and mCherry-tagged cyclin F as indicated were collected. A portion of the cell pellet was lysed directly in urea buffer to obtain the total protein, and the remained of the cell pellet was lysed in RIPA buffer to obtain a soluble fraction and that RIPA-insoluble pellet was solubilized in 7 M urea buffer to obtain an insoluble fraction. Lysates were subjected to immunoblot analysis to detect the amount of p62, cyclin F or β-actin for loading control (*n* = 4). The ratio of soluble to insoluble p62 K281R was significantly greater relative to WT p62. Analysis of replicates were carried out on the same immunoblot for comparisons. A representative immunoblot is shown with dashed line indicating cropped image from same immunoblot. Data represents mean ± SEM (*n* = 4). **p < 0.005, ns not significant, n.s. non-specific

Next we determined whether the K281 ubiquitylation affects p62 aggregation since pathologic aggregates positive for p62 and ubiquitin have been found in brain and spinal cord tissue of both familial and sporadic ALS and FTD cases [27, 34]. We investigated whether p62 ubiquitylation at K281 alters its solubility in motor neuron-like NSC-34 cells by expressing an HA-tagged K281 ubiquitylation resistant, but chemically similar, mutant of p62 (lysine 281 arginine point mutation, K281R) with mCherry-cyclin F and performed soluble and insoluble fractionation experiments. For each replicate, the fractions were immunoblotted, and levels of protein were normalized to β-actin loading control (Fig. 7). The ratio of soluble to insoluble p62 was significantly greater in the K281R mutant compared to WT expressing cells (*p* < 0.005) (Fig. 7). This data suggests that loss of ubiquitylation of p62 at K281 decreases the shift of p62 into the insoluble fraction.

### p62 Solubility is Altered by ALS and FTD-Linked Cyclin F p.S621G

**Fig. 8:**
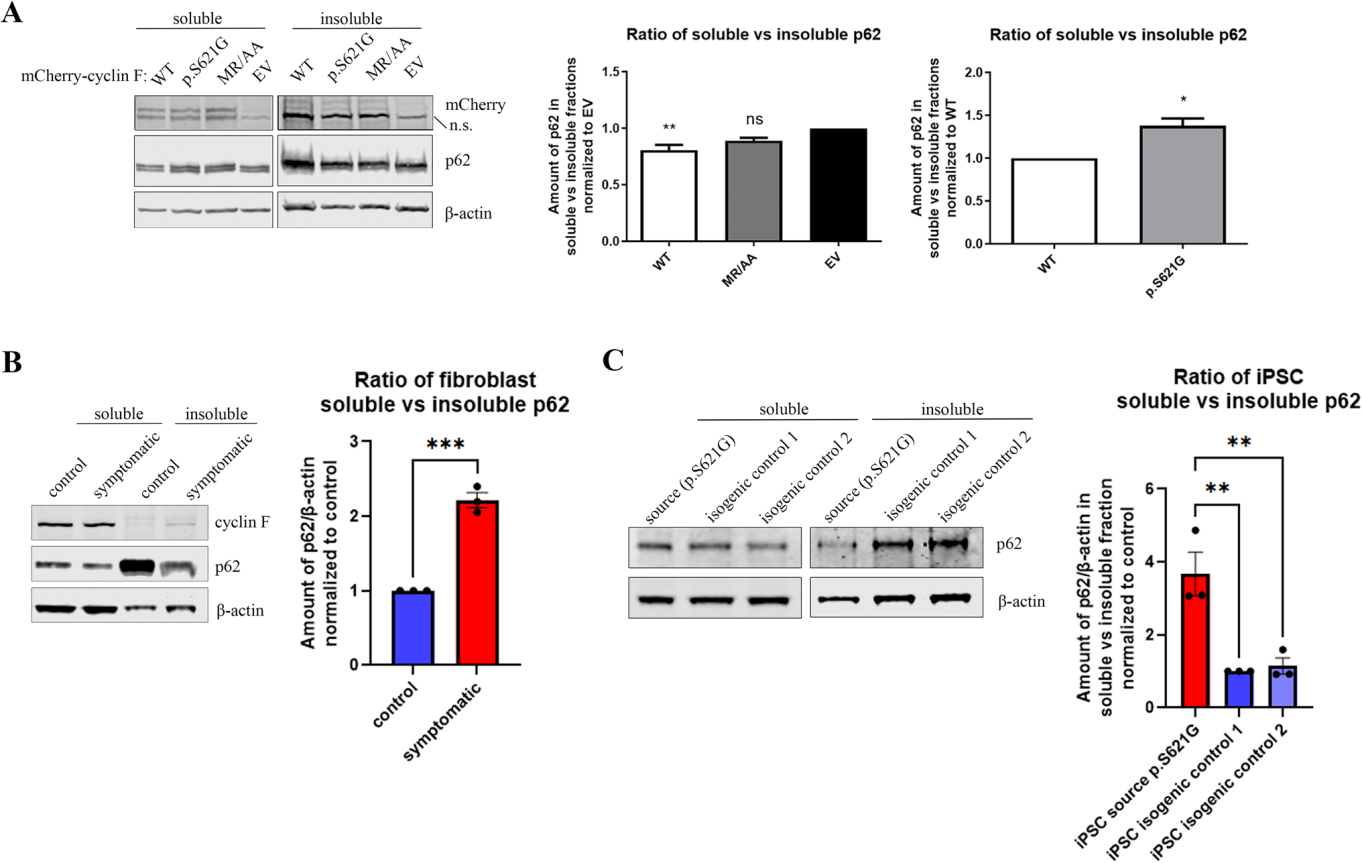
p62 solubility is altered by mutant cyclin F. Neuro2A cells expressing cyclin F WT, p.S621G, MR/AA and EV, symptomatic *CCNF* p.S621G patient fibroblasts, and *CCNF* p.S621G patient iPSC were lysed in RIPA buffer to obtain a soluble fraction and the RIPA-insoluble pellet was solubilized in 7 M urea buffer to obtain an insoluble fraction. Lysates were subjected to immunoblot analysis to detect the amount of p62, cyclin F or β-actin for loading control normalization (*n* = 3). **A**. WT and MR/AA expressing cell lysates were compared to EV transfected cell lysates to identify the effect of cyclin F expression on p62 solubility. Cyclin F p.S621G expressing cell lysates were then compared to WT expressing cell lysates to discern the effect of the p.S621G mutation on p62 solubility. **B**. Increased soluble to insoluble p62 ratio in symptomatic *CCNF* p.S621G patient fibroblasts compared to control. **C**. Increased soluble to insoluble p62 ratio in *CCNF* p.S621G patient derived iPSC compared to isogenic controls. Data represents mean ± SEM (*n* = 3). *p < 0.05, **p < 0.005, **p < 0.0005, ns not significant, n.s. non-specific

To determine the effect of ALS and FTD-linked variant cyclin F on endogenous p62 solubility, we performed fractionation of neuronal-like Neuro2A cell lysates into RIPA soluble and insoluble fractions. For each replicate, the RIPA-soluble and insoluble fractions were immunoblotted, and levels of protein were normalized to β-actin loading control. We first evaluated the effect of cyclin F (WT and binding mutant MR/AA) on p62 solubility. In comparison to mCherry-alone transfected Neuro2A cells, the ratio of soluble to insoluble p62 was significantly reduced by 20% in cyclin F WT (*p* < 0.005), but not in cyclin F MR/AA expressing cells (Fig. 8A). These data suggest that cyclin F expression promotes p62 insolubility by shifting p62 into the insoluble fraction.

To evaluate the effect of the p.S621G mutant form of cyclin F, we next compared the solubility of p62 in cyclin F WT expressing cells. Accordingly, the ratio of soluble to insoluble p62 was significantly increased by 38% in cyclin F p.S621G expressing cells compared to cyclin F WT (*p *< 0.05) (Fig. 8A). These data indicate that mutant cyclin F p.S621G expression decreased the propensity of endogenous p62 to shift into the insoluble fraction. To validate whether cyclin F p.S621G causes p62 solubility defects in ALS and FTD patient derived cells, we repeated the soluble-insoluble protein analysis in human primary fibroblast cultures from non-disease control and a symptomatic ALS and FTD-affected *CCNF* p.S621G patient that were cultured in technical triplicates. We also investigated p62 solubility in induced pluripotent stem cells (iPSC) derived from a patient with *CCNF* p.S621G compared to isogenic controls that were cultured in technical replicates [51]. In accordance with observations from Neuro2A cells, the ratio of soluble to insoluble p62 was significantly greater in the ALS and FTD patient fibroblasts (*p* < 0.0005) compared to fibroblasts from non-disease control patients (Fig. 8B). Consistently, ALS and FTD patient iPSC had increased ratio of soluble to insoluble p62 compared to the isogenic controls (*p* < 0.005) (Fig. 8C). Thus, the increased ratio of soluble to insoluble p62 is recapitulated in human primary fibroblast cells and iPSC from patients harbouring the cyclin F p.S621G mutation, indicating that mutant cyclin F aberrantly affects the solubility of p62 in disease pathogenesis.

### Cyclin F WT but not p.S621G Promotes p62 Foci Formation

**Fig. 9:**
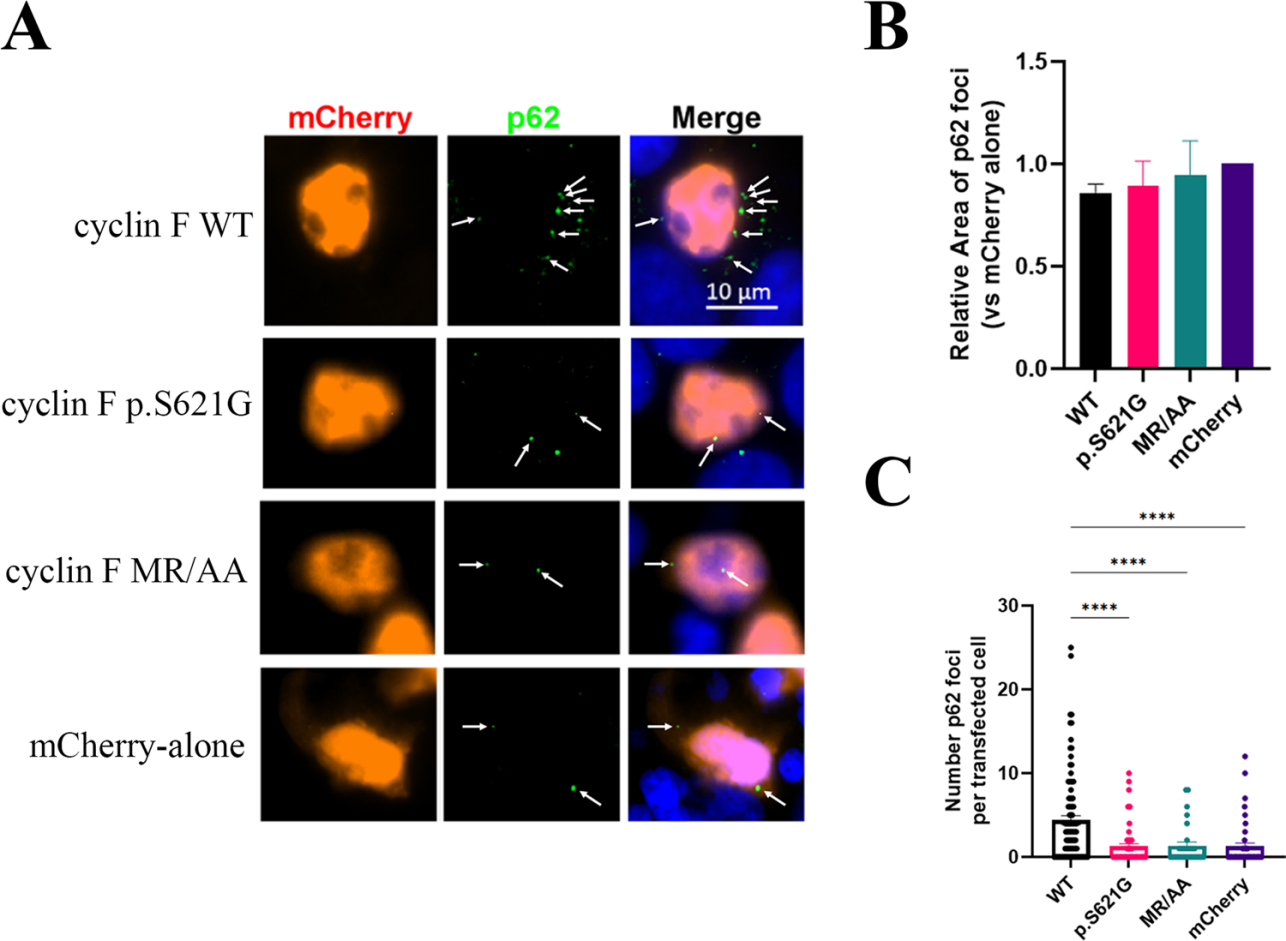
Cyclin F WT but not mutant p.S621G promotes p62 foci formation. **A**. Immunofluorescence of HEK293 cells transiently expressing variants of mCherry-tagged cyclin F (WT, p.S621G, MR/AA) and mCherry-alone to detect p62 foci (white arrows), which were analyzed using CellProfiler. **B**. The relative size (area) of the p62 foci were quantified and compared to mCherry-alone construct. **C.** Cyclin F WT, but not p.S621G, expressing cells had a significantly greater number of p62 foci than mCherry-alone construct. Only transfected cells were included for analysis (red channel) which was adjusted post-analysis for presentation and publication purposes. White arrows indicate foci of transfected cells. ****p < 0.0001

Given that cyclin F expression promoted the shift of p62 into the insoluble fraction, we questioned how this related to the formation of p62 foci and aggregates. p62 was assessed by immunofluorescence in HEK293 cells expressing mCherry-tagged cyclin F (WT, p.S621G, MR/AA) or mCherry-alone (Fig. 9A, white arrows). Interestingly we observed noticeable p62 foci in the cyclin F WT expressing cells. The size (area) and number of endogenous p62 foci were quantified in three biological replicates with at least 30 cells. No significant difference was detected in the relative area of the p62 foci between any of the groups (Fig. 9B). However, cyclin F WT expressing cells had a significantly greater number of p62 foci per cell compared with mCherry-alone and cyclin F MR/AA control constructs (*p* < 0.0001) (Fig. 9C). To ascertain the effect of the p.S621G mutation, the WT and p.S621G constructs were compared. Notably, ALS and FTD mutant cyclin F p.S621G had significantly fewer p62 foci per cell compared to cyclin F WT (*p* < 0.0001). This supports our previous finding that cyclin F WT expression but not mutant p.S621G expression promoted the propensity of p62 to aggregate into the insoluble fraction and demonstrated that the ALS and FTD linked variant of cyclin F disrupted p62 foci formation. Together, this data suggests that these p62 foci are insoluble, and are not necessarily associated to pathological aggregates in disease pathogenesis given they were observed more in WT than mutant cyclin F cells.

## Discussion

In the present study we have identified an E3 ligase, SCF^cyclin F^, that ubiquitylated p62 at K281, which regulated the shift of p62 into the insoluble fraction. Our data demonstrated that cyclin F expression led to the aggregation of p62 into the insoluble fraction which was associated with an increased number of p62 foci. Notably, this study provides further evidence for complex interplay between the two main protein clearance pathways, UPS and autophagy [36, 52–54] by demonstrating that cyclin F, which has been reported to ubiquitylate substrates for proteasomal degradation, can also affect the solubility of key autophagy-associated protein, p62. We also identified and explored the pathobiology of ALS and FTD-linked mutant cyclin F p.S621G. Notably, we demonstrated that the ALS and FTD-linked mutant cyclin F p.S621G affected p62 ubiquitylation, disrupted cyclin F-regulated p62 foci formation and aggregation. This suggests that the abnormal function of cyclin F (caused by ALS and FTD-linked mutation or other deficiency) leads to dysregulated p62 homeostasis, which might represent an underlying mechanism of ALS and FTD pathogenesis.

To date, few E3 ligases have been reported to mediate the ubiquitylation of p62, and none have affected p62 function in ALS pathogenesis. We have now shown that cyclin F canonically recognized p62 via its substrate recognition motif (^309^MRYIL^313^) and that p62 binds in a CY-dependent manner, which is known to be recognized by the MRYIL motif [6, 55]. We previously demonstrated that mutant SCF^cyclin F^ has elevated E3 ligase activity [19–21], and co-immunoprecipitation experiments in Neuro2A cells reported by Lee et al. showed that mutant cyclin F p.S621G interacted more with p62 than cyclin F WT [20]. Compared to cyclin F WT, our in vitro and in cell culture ubiquitylation assays revealed that mutant cyclin F abnormally ubiquitylated p62. Our results suggest that the elevated ubiquitin ligase activity combined with increased interaction of mutant cyclin F with p62 contributes to the increased ubiquitylation of p62.

p62 is a multifunctional scaffold protein involved in various signalling pathways including selective autophagy, inclusion body formation, protein quality control and various cellular signalling like antioxidant and anti-inflammatory pathways [34, 35]. These critical functions rely on conformational changes in p62 that are regulated by post-translational modifications including ubiquitylation. Mutant cyclin F expressing cells experience ubiquitin stress (elevated Lysine (K) 48-linked ubiquitin levels) and defective autophagy [2, 20]. From this perspective, ubiquitylation of p62 disrupts dimerization of the UBA domain of p62, allowing it to recognize polyubiquitylated cargoes for autophagy [56], which is a critical mechanism during ubiquitin stress conditions. Combined with the previous literature, our data presented here support the notion that abnormal p62 ubiquitylation by mutant cyclin F may interfere with its functions such as autophagy. While changes in p62 ubiquitylation by mutant cyclin F were modest in the cell lines, these abnormal changes are likely to underly late disease onset such as seen in ALS and FTD [57–59] leading to the significantly increased ubiquitylation of p62 observed at the end stage of disease in patient spinal cord motor neurons compared to controls. Interestingly, dysregulated p62 ubiquitylation is not restricted to ALS and FTD but has also been identified in Parkinson’s Disease pathogenesis where mutant parkin dysregulates p62 proteasomal turnover [60], supporting the idea that dysregulated p62 ubiquitylation plays a key role in the pathogenesis of neurodegenerative diseases.

Although a recent study did not find that p62 was ubiquitylated by cyclin F but detected ubiquitylated p62 in cyclin F expressing cells by diGly proteomics [61], we believe this is due to perturbations in proteostasis triggering activation of protein degradation pathways that clear p62. In the current study, proteasome and autophagy inhibitors were used to prevent the possible clearance of ubiquitylated p62 prior to detection. Moreover, the in vitro ubiquitylation assays in this study provided an isolated environment confirming p62 ubiquitylation. Differences in p62 ubiquitylation were more apparent in the post-mortem patient tissue compared to cell lines. Transient expression was used for the in culture ubiquitylation assay which potentially only represents a population of the ubiquitylated p62. The singe cell analysis of cyclin F patient neurons better reflects the effect that the cyclin F mutation had on p62 ubiquitylation, particularly at end stage of disease.

p62 can be ubiquitylated by the E3 ubiquitin ligases parkin or cullin-3 for proteasomal turnover [60, 62]. In Song et al.’s study, parkin mediated the poly-ubiquitylation of p62 mainly with K48-linked ubiquitin chains, with K63 ubiquitin modification occurring to a lesser extent [60]. It is possible that in the in vitro ubiquitylation assay in our study cyclin F utilized the WT mono-ubiquitin moieties to mediate the ligation of different ubiquitin linkages and architecture; something that remains to be explored. E3 ligase TRIM21-mediates the K63-ubiquitylation of p62 to abrogate p62 oligomerization and sequestration of proteins for p62-regulated redox homeostasis [63, 64]. Additionally, inclusion body formation and subsequent autophagy is regulated by the E3 ligase NEDD4 [46]. Although cyclin F canonically K48-ubiquitylates proteins for UPS clearance, it is evident that E3 ligase-mediated ubiquitylation of p62 promotes and regulates functions other than proteasomal turnover. In this study, we identified a ‘non-canonical’ role of cyclin F to ubiquitylate K281 on p62 and promote its insoluble aggregation, which corresponded to an increased number of p62 foci.

It has been debated whether the formation of aggregates is cytoprotective or cytotoxic causing neuronal death [65]. Both concepts are likely true depending on stage of disease and presence of underlying familial mutations. p62 plays a protective role in neurodegenerative diseases with pathogenic proteins such as TDP-43 and polyQ protein toxicity, and this protective effect was dependent on the oligomeric species of p62 and autophagic degradation [66]. The prevailing view of autophagic clearance of these aggregates is that K63-ubiquitin-tagged proteins are assembled into aggregates by p62 and other autophagy-regulating proteins, which are then engulfed by autophagosomes for degradation [67]. In fact, p62 dynamics play a critical role in the steps preceding aggregate formation. Early stages of autophagy require p62 polymerization and formation of p62 bodies [68], or cytosolic p62 inclusion bodies, which then recruit K63-linked poly-ubiquitin chains and undergo p62 phase separation [69]. Our experiments showed visible p62 foci by immunofluorescence and a low level of insoluble p62 independent of cyclin F expression. This is in line with reports that p62 self-aggregates [70], including forming fibrillar aggregates [71], and that p62 can self-oligomerize via its PB1 domain [38, 72]. Interestingly, cyclin F WT expression promoted the aggregation of p62 into the insoluble fraction which corresponded to an increased number of p62 foci per cell. Considering phosphorylation of p62 regulates p62 body formation [69], these results suggest that cyclin F mediated ubiquitylation might be another post-translational modification regulating p62 foci formation. Further, E3 ligase NEDD4 interacts with p62 to regulate inclusion body formation and subsequent autophagy [46]. It is possible that cyclin F-mediated ubiquitylation of p62 regulates p62 foci formation as a preceding step to autophagy, which requires further investigation.

It should be noted that this study utilized cell lines expressing variants of cyclin F or p62 to elucidate the mechanistic function of the protein interaction and that findings were recapitulated in disease relevant tissue. For p62 solubility, this study focused on the propensity of p62 to aggregate into the insoluble fraction given the function of the cyclin-F mediated ubiquitylation site K281 in regulating p62 solubility. Although we observed that the neuronal-like Neuro2A cells had similar levels of total p62 across cyclin F expressing lysates, we observed that patient derived fibroblasts and iPSC had varying levels of total p62. This may be attributable to the fact that fibroblasts and iPSC are maintained in culture for several weeks or months compared to cell lines, which is consistent with previous findings comparing cell lines and patient derived cells [20]. Remarkably the mutant cyclin F p.S621G abnormally ubiquitylated p62 and did not promote p62 aggregation into the insoluble fraction or the formation of more p62 foci. Instead, the number of p62 foci was significantly reduced compared to the cyclin F WT expressing cells and remained similar to cyclin F MR/AA and mCherry-alone constructs. Unexpectedly, these findings suggest that p62 foci are insoluble and that loss of the insoluble p62 foci contribute to disease pathogenesis. These findings may be a consequence of the abnormal ubiquitylation by mutant cyclin F. Notably, this is contrary to the typical notion that insoluble p62 is an indication of pathological underpinnings. Our findings may underly early disease pathogenesis, and it is possible that differences in p62 solubility occur throughout disease stage or pathology. Considering that p62 bodies are required for early stages of autophagy [68], we speculate that mutant cyclin F hinders p62’s ability to form foci, thus contributing to the underlying autophagy defects reported in p.S621G expressing neuronal-like cells and in patient fibroblasts containing the p.S621G mutation [20]. It is known that autophagy clears proteins that are associated with ALS and FTD, and our results implicate an early mechanism that is dysregulated in ALS and FTD pathogenesis. Future studies inhibiting the autophagy pathway could confirm whether mutant cyclin F plays a pathogenic role as a consequence of disrupted p62 foci formation and aggregation.

Disease-causing gene variants in *CCNF* in familial and sporadic ALS and FTD have been linked to dysregulation of ALS- and FTD-associated proteins such as TDP-43 [22], SFPQ [23], VCP [25] and indirectly, p62 [20]. Our group recently found that mutant cyclin F p.S621G aberrantly K48-ubiquitylates ALS-associated protein TDP-43 [22], and have now shown here that cyclin F p.S621G aberrantly ubiquitylates and dysregulates p62 as well. Since increased p62 levels alter TDP-43 dynamics, it would also be interesting to elucidate whether cyclin F-mediated ubiquitylation of p62 also contributes to TDP-43 pathology [73].

p62 converges multiple ALS and FTD-associated signalling pathways and proteins (as recently reviewed in [34]). As such, abnormal p62 regulation by mutant cyclin F may contribute to neuronal toxicity via multiple mechanisms. For example, our group reported that mutant cyclin F triggers neuronal apoptosis in cells and zebrafish [21]. Further, it has been shown that interference with p62 function increases cell death in Huntingtin disease pathogenesis [68]. Thus, future research to evaluate whether cyclin F-mediated ubiquitylation disrupts p62 function in the apoptosis pathway in relation to ALS and FTD pathogenesis warrants further investigation.

Overall, our study demonstrates that p62 is a novel substrate of cyclin F. We provide a mechanistic link between cyclin F and p62 regulation, with implications for ALS and FTD pathogenesis. Importantly, we found that mutant cyclin F p.S621G abnormally ubiquitylates p62, and leads to decreased p62 foci formation and aberrant solubility, which are events that have been linked to neurodegenerative diseases including ALS and FTD. Together, our results show that an ALS and FTD linked mutation in cyclin F can lead to p62 dysregulation, supporting the notion that abnormal p62 mediated regulation of protein clearance or cell death pathways may underly ALS and FTD pathogenesis.

## Materials and Methods

### Immunological Reagents

Commercially available antibodies used in this study are available in Supplementary Table 1, with corresponding experiment dilutions and catalogue numbers.

### Plasmids and Cloning

Expression constructs encoding wild type and p.S621G *CCNF* cDNA fused to an N-terminal mCherry fluorophore were used as described previously [2]. The MR/AA (M309A/R310A) and LP/AA (L35A/P36A) *CCNF* mutants were subcloned into pmCherry-C1 vector. Wild type *CCNF* cDNA fused to a N-terminal Flag-tag was also cloned into a pcDNA3.1 vector (GenScript).

To create the *CCNF* deletion (∆) constructs (∆PEST, amino acids ∆582–766; ∆CyclinBox, amino acids ∆292–528; ∆CyclinN, amino acids ∆292–405; ∆CyclinC, amino acids ∆408–528; and ∆292–766, amino acids ∆292–766) with an N-terminal mCherry fluorophore, *CCNF* cDNA was first inserted into the pGEM-T vector and then *CCNF* deletion fragments were subcloned pmCherry-C1 vector. A validation PCR demonstrated that sub-cloning into the pmCherry-C1 vector was successful for all deletion fragments. Sequencing of the nucleotides flanking the deleted region for the *CCNF* deletion constructs confirmed the expected sequences.

Wild type *SQSTM1/p62* cDNA fused to a N-terminal HA-tag was cloned into a pcDNA3.1 vector, which also served for mutagenesis to create the p62 RxL/AxA constructs (GenScript). pCI-His-hUbi was a gift from Astar Winoto (Addgene plasmid # 31,815; http://n2t.net/addgene:31815; RRID:Addgene_31815) [74].

### Cell Lines and Culture Conditions

HEK293, Neuro2A, NSC-43 cells and fibroblasts were grown in DMEM supplemented with 10% (v/v) fetal bovine serum (FBS, Sigma-Alrich) and maintained as previously described [20, 22, 75, 76]. Induced pluripotent stem cells were maintained as previously described [51]. Cells were maintained in an incubator (37^◦^C, 5% CO_2_ and 95% humidity). All cell lines were routinely tested for mycoplasma using MycoAlert Mycoplasma Detection Kit (Lonza). Cells were harvested into ice-cold NP-40 lysis buffer supplemented with 1X protease inhibitor cocktail (Roche) and 1X phosphatase inhibitor cocktail (Roche).

### Transient Transfections and Drug Treatments

All transient transfections were conducted using Lipofectamine2000 (Thermo Fisher Scientific) as per manufacturer’s protocol. Up to a total of 0.6 µg or 7 µg of DNA was used for transfections in 12 wells and T75 flasks, respectively. Protein expression was assessed via immunoblot or immunofluorescence analysis.

Proteasome inhibitor MG132 (5 µM, Selleckchem) and autophagy inhibitor, chloroquine (CQ) (100 µM, 7 h, Selleckchem) were used to treat HEK293 cells for the in vivo ubiquitylation assay. Control for drug treatment included incubation with DMSO (vehicle). We subjected cells transiently expressing cyclin F, p62, and ubiquitin for 24 h to MG132 and CQ for 7 h. Cells were then lysed in urea lysis buffer supplemented with 1X protease inhibitor cocktail and 1X phosphatase inhibitor cocktail for immunoblot analysis and immunoprecipitation under denaturing conditions.

### Total Cell Lysis

Cells were harvested by scraping into ice-cold PBS. Flasks were rinsed three time with ice-cold PBS, with each rinse collectively added to the collected cells. Centrifugation at 1,000xg at 4 °C for 5 min was conducted to collect the cell pellet. Supernatants were discarded. Pellets were incubated on ice for 20 min in NP-40 lysis buffer (1% NP-40, 50 mM Tris–HCl, 150 mM NaCl, 2 mM EDTA, 10 mM NEM), RIPA lysis buffer (0.25% SDC, 1% NP-40, 50 mM Tris–HCl, 150 mM NaCl, 1 mM EDTA), or urea lysis buffer (7 M urea, 2 M Thiourea, 4% CHAPS, 30 mM Tris) supplemented with 1X protease and 1X phosphatase inhibitor with intermittent vortexing before ultrasonication with Sonic Ruptor 250 at 50% power and pulser settings set to 30% for 10 pulses each. Centrifugation at 14,000xg for 15 min at 4 °C. The supernatant containing cellular protein was collected for assessing protein concentration and experimental use.

### Proximity Ligation Immunofluorescence Assay

Duolink in situ proximity ligation assay (PLA) [48] (Sigma-Aldrich) was used per manufacturer’s protocol. To prepare HEK293 cells for PLA analysis, cells were grown asynchronously on coverslips in 12 well plates to approximately 50–70% confluency, fixed in 4% paraformaldehyde (PFA) in PBS for 20 min and rinsed three times with PBS. Cells were then permeabilized with 0.2% Triton X-100 in PBS for 20 min, blocked per Duolink PLA blocking buffer, followed by incubation with primary antibodies anti-Cyclin F (mouse), anti-p62 (rabbit), or anti-cdc6 (rabbit) followed by PLA analysis, prepared for fluorescence microscopy and imaged using Texas Red filter (Zeiss). Upon recognition of primary antibodies, secondary antibodies (PLA probes) that contain a unique DNA strand hybridise to make circular DNA when in close proximity (< 40 nm). PLA utilizes rolling circle DNA amplification that can be visualized by fluorescently labelled complementary oligonucleotide probes. The result is fluorescent foci at spots of proximity (the endogenous protein interaction) that can be visualized by fluorescence microscopy [48].

Primary antibodies were incubated for 1 h at room temperature. PLA probes (rabbit and mouse) were then added for 1 h at 37 °C, followed by ligation for 30 min at 37 °C and amplification for 100 min at 37 °C. Coverslips were mounted on slides with ProLong Gold Antifade Mountant with DAPI (Thermo Fisher Scientific) for fluorescence microscopy imaging using Texas Red filter to visualise the red fluorescent PLA foci. Negative technical controls included omission of all primary antibodies (secondary antibody PLA Probes only) and omission of each primary antibody separately (anti-cyclin F only, anti-p62 only). Negative biological control included IgG control, and positive biological control included known cyclin F protein interactor, cdc6 (anti-cyclin F with anti-cdc6). Three biological replicates were repeated whereby the mean was compared to the cyclin F only and p62 only controls. Quantification of PLA foci was conducted using a python script (https://github.com/doc78/PLA-Analysis), with a pipeline to pre-process the images and then a Watershed algorithm to obtain the final segmentation. In brief, the images were converted to black and white, with white pixels representing the fluorescent red foci, and then white spots (foci) were outlined and quantified. Foci counts were confirmed by counting of randomly selected images.

### Immunofluorescence Microscopy

Cells were fixed on coverslips using 4% paraformaldehyde (PFA) in PBS for 20 min and rinsed three times with PBS. Cells were then permeabilized using 0.2% Triton X-100 in PBS for 20 min followed by blocking with 1% BSA, 22.52 mg/mL glycine in 0.1% Triton X-100 in PBS for 30 min. Antibodies were diluted in PBS-T (PBS-Tween 20, 0.05%) and 2% NGS. Primary antibody was added and incubated at room temperature for 2 h. Coverslips were gently washed three times with PBS for a total of 15 min followed by incubation with secondary antibody for 1 h at room temperature in the dark. Secondary antibody anti-mouse AlexaFluor488 was diluted 1:200 and used to detect p62. Coverslips were mounted on glass slides using ProLong Gold Antifade Mountant with DAPI (Thermo Fisher Scientific). Staining and fluorescent tags were visualized and captured using Zeiss Fluorescence microscope. Controls for specificity included secondary antibody staining without primary antibody (data not shown). Three biological replicates were completed with at least 30 cells. P62 foci in only mCherry-cyclin F or mCherry-alone transfected cells were analyzed using CellProfiler.

### mcherry and HA Immunoprecipitations

For immunoprecipitation, HEK293 and Neuro2A cells were lysed in ice-cold NP-40 lysis buffer supplemented with 1X protease inhibitor cocktail and 1X phosphatase inhibitor cocktail. Resuspended cells were chilled on ice for 15 min with intermittent vortexing (3x), then sonicated 10 × using Sonic Ruptor 250 at 50% power and pulser settings set to 30%, and centrifuged at 14,000xg for 20 min to remove cellular debris. Protein concentration was estimated using Pierce™ BCA Protein Assay Kit (Thermo Fisher Scientific). Immunoprecipitations were carried out using approximately 500 µg of cellular protein and 20 µL of RFP-Trap® magnetic beads (Chromotek) to recognize the mCherry-tag, or anti-HA magnetically couple beads (Pierce, Thermo Fisher Scientific) to recognize the HA-tag as per manufacturer’s instructions. For co-immunoprecipitation experiments, non-specific binding controls included incubating beads with lysates transfected with mCherry-tag or pcDNA3.1 empty vector (EV), as well as incubation with just NP-40 lysis buffer (instead of lysate). Magnetic beads were collected using a magnet and washed three times in NP-40 lysis buffer. Elution was performed with 2X sample buffer was added to samples and boiled at 95^◦^C for 10 min, then analyzed by immunoblot.

### In Vitro Ubiquitylation Assay

SCF^cyclin F^-mediated ubiquitylation of p62 was determined using in vitro ubiquitylation assays. HEK293 cells were transiently transfected with mCherry-tagged Cyclin F WT, ALS and FTD-mutant p.S621G, reduced activity mutant (L35A/P36A, referred to as LP/AA), or mCherry tag alone using Lipofectamine 2000 according to manufacturer’s instructions. Cells were lysed in NP-40 lysis buffer supplemented with 1X protease inhibitor cocktail and 1X phosphatase inhibitor cocktail. mCherry tagged Cyclin F was immunoprecipitated (500 µg) using RFP Trap magnetic beads as per previous immunoprecipitation protocol to ensure pull down of the SCF complex (Skp1, Cul1, Rbx1). Immunoblotting confirmed the SCF complex was intact and suitable for use in the in vitro ubiquitylation assay. As a control, the F-Box mutant, Cyclin F (L35P/A36A) served as a catalytically inactive control which does not bind to Skp1 in the SCF complex but retains binding to its substrates and therefore the LP/AA mutant is unable to promote the ubiquitylation [9, 12]. The LP/AA mutant has been used as an appropriate control in Cyclin F ubiquitylation studies which was first demonstrated by D’Angiolella et al. [7], and we confirmed the LP/AA construct generated in our lab and used in this study was consistent [22, 23].

To assess the ubiquitylation status of p62 by cyclin F, immunoprecipitated cyclin F was washed three times in NP-40 lysis buffer and then twice in ubiquitylation assay buffer (100 mM Tris–HCl, 10 mM MgCl_2_, 0.2 mM DTT, pH 8.0). The in vitro assay was performed in a volume of 50 µL containing 5 µg His-tagged recombinant human SQSTM1/p62 protein (ab95320, abcam), and 10 nM E1 (UBA1; BML-UW9410, Enzo Life Sciences) and 100 nM E2 (UBE2D3; UB-42899, Life Sensors) conjugating enzymes, 10 µg/mL of biotinylated-tagged ubiquitin (BML-UW8705, Enzo Life Sciences), with or without 1.6 mM ATP, incubated with agitation (800 rpm) for 2 h at room temperature. Half the sample was analyzed by immunoblot. The recombinant p62 protein was a protein fragment from amino acids 85 to 440. This was used as full length recombinant p62 protein was not available.

### In Vivo Ubiquitylation Assay

HEK293 and Neuro2A cells expressing constructs of FLAG-cyclin F, HA-p62 and His-ubiquitin [74] for 24 h were lysed in denaturing urea lysis buffer. Ubiquitylated proteins were immunoprecipitated using a 50% slurry of Ni–NTA resin agarose beads under denaturing conditions for 30 min at room temperature. Samples were centrifuged at 15,000xg for 10 s and supernatant was discarded. The resin was washed twice with urea lysis buffer (pH 6.3). This was repeated three times. The ubiquitylated proteins were eluted from the beads using urea lysis buffer (pH 4.5), centrifuged for 10 s at 15,000xg. The supernatant was collected into a new tube. This was repeated three times, and the supernatants were combined. Three biological replicates were completed. Immunoblot analysis was used to determine ubiquitylation status.

### Soluble/Insoluble Protein Fractionation

Transiently transfected Neuro2A cells expressing variants of cyclin F were washed 3 × with ice-cold PBS and the cell pellet was collected by centrifugation. Cells were treated with DMSO for drug treatment comparison studies. The cell pellets of Neuro2A cells, patient fibroblasts and iPSC were resuspended in 200–300 µL ice-cold RIPA buffer containing 1X protease inhibitor cocktail and 1X phosphatase inhibitor cocktail. Resuspended cells were chilled on ice for 15 min with intermittent vortexing (3x), then sonicated 10 × using Sonic Ruptor 250 at 50% power and pulser settings set to 30%. Lysates were ultracentrifuged (Beckman Coulter) at 100,000xg for 30 min at 4^◦^C. The resulting supernatant was collected as the RIPA-soluble fraction. The remaining pellet was washed 3 × with RIPA buffer and resuspended in RIPA buffer prior to repeat sonication and ultracentrifugation. This step was repeated 3 × to remove as many RIPA soluble proteins as possible. The supernatant was discarded, and the pellet was resuspended in 50–100 µL urea buffer (7 M urea, 2 M Thiourea, 4% CHAPS, 30 mM Tris) followed by identical sonication and ultracentrifugation. The supernatant collected was urea-soluble which represents the RIPA-insoluble fraction. Fractions were then subjected to immunoblot analysis.

### SDS Page and Immunoblot Analysis

Protein concentration was estimated using Pierce™ BCA Protein Assay Kit (Thermo Fisher Scientific). Equal amounts of protein were separated using 4–15% Tris–Glycine (Bio-Rad) precast SDS-PAGE gel, using Tris–Glycine-SDS buffer, typically with 10 µg of protein. Proteins were transferred onto a nitrocellulose membrane using the Bio-Rad Trans-blot Turbo Transfer System (1.3 A, 25 V, 10 min). Membranes were blocked in Odyssey® Blocking Buffer (LI-COR) in TBS for 1 h prior to incubation with primary antibody overnight at 4^◦^C or for 1 h at room temperature (RT). Membranes were washed 3 × with TBS-T for 15 min prior to adding fluorescently labelled IRDye® 680RD goat anti-rabbit or IRDye® 800CW goat anti-mouse IgG secondary antibodies (1:10,000, LI-COR) each for 30 min at RT. Antibody against β-actin was used to normalize the protein of interest prior to making comparisons. Immunoblots were imaged using LI-COR Odyssey CLx imaging system at 700 nm and 800 nm wavelengths, respectively, and quantified by LI-COR analysis software.

### Reverse Phase Liquid Chromatography Mass Spectrometry (LC–MS/MS)

LC–MS/MS analysis was carried out as recently described [21] with slight modifications.

### Trypsin Digestion for LC–MS/MS

Samples were separated by SDS-PAGE gel electrophoresis (BioRad) for in-gel reduction with 10 mM DTT, alkylation with 20 mM IAA and trypsin digestion (1:50 enzyme:protein) overnight at 37 °C (V5111, Promega). Extracted peptides were lyophilized and then resuspended in 0.1% formic acid (FA) for desalting using C18 OMIX tips (A57003100K, Agilent). Samples were lyophilized again and resuspended in 0.1% FA, bath sonicated for 20 min, and then centrifuged at 14,000xg for 15 min to remove insoluble debris, and the supernatants (clarified peptides) were aliquoted into vials and then analyzed by LC–MS/MS.

### LC–MS/MS

The Ultimate 3000 nanoLC (Thermo Fisher Scientific) fitted with the Acclaim PepMap RSLC column particle size of 2 μm, diameter of 0.075 mm and length of 150 mm (Thermo Fisher Scientific) was used, employing a 60-min gradient (2–80% v/v ACN, 0.1% v/v FA) running at a flow rate of 300 nl/min to separate peptides. Subsequently, eluted peptides were ionized into the Q Exactive Plus mass spectrometer (Thermo Fisher Scientific) that had an electrospray source fitted with an emitter tip 10 μm (New Objective) and maintained at 1.6 kV electrospray voltage. Capillary temperature was set to 250 °C. A data-dependent “Top 10” method operating in FT acquisition mode with HCD fragmentation was used for MS/MS fragmentation to select precursor ions. On the Q Exactive Plus, FT-MS analysis was carried out at 70,000 resolution with an AGC target of 1 × 10^6^ ions in full MS and a maximum injection time of 30 ms; And MS/MS scans were carried out at 17,500 resolution with an AGC target of 2 × 10^4^ ions with maximum injection times set to 50 ms. The ion selection threshold was set to 25,000 counts to trigger MS/MS fragmentation. HCD fragmentation was performed using an isolation width of 2.0 Da with a normalized collision energy of 27.

Raw files were searched with Proteome Discoverer 2.4 (Thermo Fisher Scientific) against Uniprot FASTA database incorporating the Sequest search algorithm. Search parameters accounted for 20 ppm precursor ion tolerance and 0.1 Da MS/MS fragment ion tolerance for FT-MS and HCD fragmentation. The search allowed for static modifications of cysteine carbamidomethylation and variable modifications of methionine oxidation, asparginine and glutamine deamidation, acetylated N-terminal residues and GlyGly on lysine residues. Two missed cleavages were allowed for. The data was processed through Percolator for estimation of false discovery rates. Protein identifications were validated employing a q-value of 0.01. Label-free quantitation (LFQ) using intensity-based quantification was carried out using default parameters in Proteome Discoverer 2.4 incorporating “unique” peptides for quantification and “total peptide amount” normalisation.

### *CCNF* Patient Post-Mortem Spinal Cord Tissue Staining

Five-µm thickness post-mortem formalin-fixed paraffin-embedded cervical spinal cord tissue sections from two ALS patients carrying either the p.S621G or the p.S195R mutation were obtained from Sydney Brain Bank and the CIEN Tissue Bank, respectively. Neurologically normal control patient cervical spinal cord tissues were obtained from Sydney Brain Bank. In compliance with the brain bank's protocols, an informed consent for brain donation was obtained for both Brain Banks. Demographic information of cases is listed in Table 1. To examine the interaction between p62 and ubiquitin, dual-immunofluorescence was performed using methods described in McCann et al., 2020 [77]. Briefly, tissue sections were deparaffinized with xylene and rehydrated with a series of decreasing concentration of ethanol solution and water. Heat-induced antigen retrieval was performed in 10 mM citrate buffer, pH 6.0 (Sigma-Aldrich). Non-specific antibody binding was blocked using 5% normal goat serum. p62 and ubiquitin was detected using primary antibody against p62 (Merck MABN130, 1:50 dilution) and ubiquitin (Dako Z0458, 1:100 dilution), followed by Alexa Fluor 488 and 555 conjugated secondary antibodies (1:250 dilution). NeuroTrace 640/660 Deep-Red Fluorescent Nissl Stain (ThermoFisher Scientific N21483) was used to label neurons.

**Table 1:** Demographic information of cases used in this study

Identification	Origin	Sex	Age of onset (years)	Duration (years)	Age at death
Control	Asian	Male	N/A	N/A	69
Control	European	Male	N/A	N/A	61
Control	European	Female	N/A	N/A	59
Control	European	Female	N/A	N/A	62
Cyclin F (p.S195R)	Spanish	Female	54	2	56
Cyclin F (p.S621G)	European	Male	62	3	65

### Post-Mortem Tissue Image Acquisition and Analysis

Z-stack images of motor neurons from the ventral horn region of the spinal cord were acquired using ZEISS LSM 880 inverted confocal laser-scanning microscope with a 63 × lens (NA = 1.4) with ZEN Black software. At least eight motor neurons were imaged from cyclin F patients and three to four motor neurons were imaged from each control. Images were subject to deconvolution using Imaris.

Image analysis was performed in Fiji (ImageJ 1.53t). The Auto Threshold function (Fiji) was applied to three randomly selected images for each channel. The thresholds with the best signal-to-noise ratio were selected and applied to all images.. Neurons were then selected using Freehand Selection Tool to create regions of interest. Colocalization and area measurements were finally performed using the BIOP Just Another Colocalization Plugin (JACoP) https://c4science.ch/w/bioimaging_and_optics_platform_biop/image-processing/imagej_tools/jacop_b/ [78, 79].

### Statistical Analysis

Statistical analysis was performed using by GraphPad Prism 9.0 with significance considered as *p *< 0.05. At least three biological replicates (*n* ≥ 3) were conducted for each experiment. Data are presented as mean value ± standard error of the mean (SEM). One-way ANOVA, using the Brown-Forsythe correction for unequal variance followed by Dunnett’s *post-hoc* comparison was used. The significance threshold was set at *p* = 0.05. Comparisons made between two samples (i.e. WT and mutant) were conducted using an unpaired t-test with Welch’s correction for unequal variance.

## Supplementary Information

Below is the link to the electronic supplementary material.

Supplementary file1 (DOCX 15 KB)

## Data Availability

All datasets generated and analyzed during this study are included in this published article and its supplementary information files. Materials are available upon request.

## Abbreviations

ALS: Amyotrophic lateral sclerosis
FTD: Frontotemporal dementia
*CCNF*: Cyclin F
SCF: Skp1-Cul1-F-box complex
*SQSTM1*/p62: Sequestosome-1/p62
MRYIL: Met-Arg-Tyr-Ile-Leu amino acids
CY: Cyclin binding motif
UPS: Ubiquitin proteasome system
PB1: Phox-BEM1 domain
PEST: Region rich in proline, glutamic acid, serine, and threonine
UBA: Ubiquitin associated domain
RxL: Arginine-any amino acid-Leucine
RxI: Arginine-any amino acid-Isoleucine
PLA: Proximity ligation assay
IgG: Immunoglobulin G
RFP: Red fluorescent protein
HA: Hemagglutinin tag
FLAG: Tag with peptide sequence DYKDDDDK
NP40: Nonidet P40
IP: Immunoprecipitate
DAPI: 4′,6-Diamidino-2-phenylindole
ATP: Adenosine triphosphate
NuSAP1: Nucleolar and spindle-associated protein 1
CDC6: Cell division cycle 6
SLBP: Stem-loop binding protein
RRM2: Ribonucleotide reductase M2
CP110: Centriolar coiled coil protein 110
E2Fs: Transcription factors
EXO1: Exonuclease 1
Chd1/fzr1: Cadherin-1
RBPJ: Recombinant signal binding protein for immunoglobulin kappa J region
SIRT5: NAD-dependent protein deacylase sirtuin-5
TDP-43: Tar DNA-binding protein of 43 kDa
VCP: Valosin-containing protein
p.S621G: Serine621Glycine point mutation
SFPQ: Splicing factor proline and glutamine rich
C9ORF72: Chromosome 9 open reading frame 72
SOD1: Superoxide dismutase 1
TBK1: TANK binding kinase 1
FUS: Fused in sarcoma
HEK293(T): Human embryonic kidney cell lines
WT: Wild type
nm: Nanometers
SKP1: S-Phase Kinase Associated Protein 1
EV: Empty vector
CQ: Chloroquine
TRIM21: Tripartite motif containing-21
NEDD4: Neuronal precursor cell-expressed developmentally downregulated 4
K: Lysine
NSC-34: Motor neuron like cell line
iPSC: Induced pluripotent stem cells
