# Supplementary material for: The E3 Ubiquitin Ligase SCF Cyclin F Promotes Sequestosome-1/p62 Insolubility and Foci Formation and is Dysregulated in ALS and FTD Pathogenesis
Source: Mol Neurobiol. 2023 May 27;60(9):5034–54. doi: 10.1007/s12035-023-03355-2 (PMC10415446; doi:10.1007/s12035-023-03355-2)
Supplement: Supplementary file 1 — Supplementary file1 (DOCX 15 KB) [file 12035_2023_3355_MOESM1_ESM.docx]

**SUPPLEMENTARY**

**Table 1.** List of antibodies used.

| Target | Antibody species | Dilution | Catalogue number, Company |
| --- | --- | --- | --- |
| Cyclin F (C-20) | rabbit polyclonal | IB: 1:500 | sc-952, Santa Cruz Biotechnology |
| Cyclin F (B-6) | mouse monoclonal | PLA: 1:100 | sc-515207, Santa Cruz Biotechnology |
| mCherry | rabbit polyclonal | IB: 1:1000 | NBP2-43727, Novus Biologicals |
| p62/SQSTM1 [GT1478] | mouse monoclonal | IB: 1:1000 | GTX629890, GeneTex |
| p62/SQSTM1 | rabbit polyclonal | PLA: 1:1000 | P0067, Sigma-Aldrich |
| p62/SQSTM1 | mouse monoclonal | IB: 1:1000 | ab56416, abcam |
| FLAG M2 | mouse monoclonal | IB: 1:1000 | F1804, Sigma-Aldrich |
| β-actin | mouse monoclonal | IB: 1:20,000 | A1978, Sigma-Aldrich |
| HA (.11) | mouse monoclonal | IB: 1:2,000, IF: 1:200 | 901501, BioLegend |
| Cdc6 | rabbit polyclonal | PLA: 1:250 | PA5-77901, Thermo Fisher Scientific |
| Rbx1 (D3J5I) | rabbit monoclonal | IB: 1:1,000 | 11922, Cell Signaling Technology |
| Skp1 [EPR3304] | rabbit monoclonal | IB: 1:2,000 | 76502, abcam |
| Cul1 | mouse monoclonal | IB: 1:1,000 | sc-17775, Santa Cruz Biotechnology |
| AlexaFluor488 | mouse secondary | IF: 1:200 | A11001, Thermo Fisher Scientific |
| IRDye® 800CW | mouse secondary | IB: 1:10,000 | LI-COR Biosciences |
| IRDye® 680RD | rabbit secondary | IB: 1:10,000 | LI-COR Biosciences |
| RFP-Trap® Magnetic Agarose |  |  | Rtma-20, Chromotek |
| Pierce™ Anti-HA Magnetic Beads |  |  | 88836, Pierce Thermo Scientific |
| Ni-NTA Agarose Beads |  |  | Qiagen |
